# Bone and body composition by DXA in girls with precocious puberty, premature thelarche, and prepubertal controls

**DOI:** 10.1210/jendso/bvag023

**Published:** 2026-01-30

**Authors:** Daniela Fava, Amanda Casirati, Alessia Angelelli, Maria Grazia Calevo, Carlotta Pepino, Alessia Pepe, Nadia Gabriella Maiorano, Lucia Acquarone, Agnese Repetto, Chiara Santucci, Claudia Caridi, Caterina Tedesco, Flavia Napoli, Anna Elsa Maria Allegri, Giuseppa Patti, Roberto Gastaldi, Marta Panciroli, Alessandro Naim, Mohamad Maghnie, Natascia Di Iorgi

**Affiliations:** Pediatric Endocrinology Unit, Department of Pediatrics, IRCCS Istituto Giannina Gaslini, Genoa 16147, Italy; Pediatric Endocrinology Unit, Department of Pediatrics, IRCCS Istituto Giannina Gaslini, Genoa 16147, Italy; Pediatric Endocrinology Unit, Department of Pediatrics, IRCCS Istituto Giannina Gaslini, Genoa 16147, Italy; Epidemiology and Biostatistics Unit, Scientific Directorate, IRCCS Istituto Giannina Gaslini, Genoa 16147, Italy; Pediatric Endocrinology Unit, Department of Pediatrics, IRCCS Istituto Giannina Gaslini, Genoa 16147, Italy; Department of Neuroscience, Rehabilitation, Ophthalmology, Genetics, Maternal and Child Health, University of Genoa, Genoa 16132, Italy; Department of Neuroscience, Rehabilitation, Ophthalmology, Genetics, Maternal and Child Health, University of Genoa, Genoa 16132, Italy; Department of Neuroscience, Rehabilitation, Ophthalmology, Genetics, Maternal and Child Health, University of Genoa, Genoa 16132, Italy; Department of Neuroscience, Rehabilitation, Ophthalmology, Genetics, Maternal and Child Health, University of Genoa, Genoa 16132, Italy; Department of Neuroscience, Rehabilitation, Ophthalmology, Genetics, Maternal and Child Health, University of Genoa, Genoa 16132, Italy; Department of Neuroscience, Rehabilitation, Ophthalmology, Genetics, Maternal and Child Health, University of Genoa, Genoa 16132, Italy; Pediatric Endocrinology Unit, Department of Pediatrics, IRCCS Istituto Giannina Gaslini, Genoa 16147, Italy; Pediatric Endocrinology Unit, Department of Pediatrics, IRCCS Istituto Giannina Gaslini, Genoa 16147, Italy; Pediatric Endocrinology Unit, Department of Pediatrics, IRCCS Istituto Giannina Gaslini, Genoa 16147, Italy; Pediatric Endocrinology Unit, Department of Pediatrics, IRCCS Istituto Giannina Gaslini, Genoa 16147, Italy; Department of Neuroscience, Rehabilitation, Ophthalmology, Genetics, Maternal and Child Health, University of Genoa, Genoa 16132, Italy; Pediatric Endocrinology Unit, Department of Pediatrics, IRCCS Istituto Giannina Gaslini, Genoa 16147, Italy; Department of Neuroscience, Rehabilitation, Ophthalmology, Genetics, Maternal and Child Health, University of Genoa, Genoa 16132, Italy; Department of Neuroscience, Rehabilitation, Ophthalmology, Genetics, Maternal and Child Health, University of Genoa, Genoa 16132, Italy; Pediatric Endocrinology Unit, Department of Pediatrics, IRCCS Istituto Giannina Gaslini, Genoa 16147, Italy; Department of Neuroscience, Rehabilitation, Ophthalmology, Genetics, Maternal and Child Health, University of Genoa, Genoa 16132, Italy; Pediatric Endocrinology Unit, Department of Pediatrics, IRCCS Istituto Giannina Gaslini, Genoa 16147, Italy; Department of Neuroscience, Rehabilitation, Ophthalmology, Genetics, Maternal and Child Health, University of Genoa, Genoa 16132, Italy

**Keywords:** precocious puberty, premature thelarche, body composition, bone mineral density, trabecular bone score, dual-energy X-ray absorptiometry

## Abstract

**Context:**

Lumbar spine bone mineral density (BMD) rises sharply during puberty; earlier onset, as in central precocious puberty (CPP), may accelerate skeletal maturation and modify bone accrual.

**Objective:**

To assess bone and body composition in girls with CPP/early puberty (EP), premature thelarche (PT), and prepubertal controls (PC).

**Patients and methods:**

We analyzed 184 girls aged 5-9 years with suspected CPP/EP (108 CPP/EP, 76 PT) and 47 PC. DXA assessed L1-L4 and total body less head (TBLH) BMD, bone mineral content (BMC), and body composition. Derived measures included bone mineral apparent density (BMAD), trabecular bone score (TBS), android–gynoid fat ratio (A/G), fat mass index (FMI), and fat-free mass index (FFMI).

**Results:**

Group CPP/EP showed greater height, BMI SDS, bone age (BA), FM, lean mass, and FFMI than controls, with higher L1-L4 BMD (*P* < .001) and a trend for higher ΔBMD L1-L4–TBLH *Z*-score (*P* = .07); L1-L4 BMAD *Z*-scores were similar. Versus Group PT, Group CPP/EP had higher Δheight-target height SDS (*P* = .005), BA (*P* < .001), and lean mass (*P* = .03); Group PT had higher A/G (*P* = .02) and TBS (*P* = .03). Within Group PT, girls with pubarche had higher height, BMI SDS, BA, FMI, A/G (*P* = .02) and L1-L4 BMAD *Z*-scores (*P* = .01).

**Conclusion:**

Both CPP/EP and PT showed higher fat and lean mass than controls, with PT marked by greater central adiposity. Only overweight/obesity, and pubarche onset in PT, were associated with increased L1-L4 BMAD *Z*-scores. DXA provides additional insight into body composition and bone accrual in girls with early pubertal signs.

Puberty represents a critical window for skeletal development, marked by rapid increases in bone mineral density (BMD) and bone mineral content (BMC) [1]. During this period, more than 60% of peak bone mass is acquired, with the most substantial gains typically occurring between the ages of 11 and 15 years, particularly during Tanner stages III and IV [2-6]. Several studies have reported that BMD increases significantly with age and pubertal progression, with the lumbar spine (L2 to L4) and femoral neck showing the greatest rates of mineral accumulation [3-6]. By the age of 14-15 years, girls often reach values close to peak bone mass, up to 99.2% at the lumbar spine and 105.1% at the femoral neck [4]. After this point, particularly 2 to 4 years post-menarche, the rate of bone accrual declines sharply [4].

The timing of pubertal onset plays a key role in determining peak bone mass [7-9]. Girls who begin puberty earlier tend to achieve higher BMD and BMC than those with delayed pubertal development [10-12]. Specifically, each year of earlier onset has been associated with a 5% increase in BMC and a 2.5% increase in BMD at skeletal maturity [10]. These observations highlight the importance of hormonal timing for optimal skeletal outcomes [6, 13]. Estrogens are pivotal in promoting bone growth, enhancing mineralization, and inducing epiphyseal closure. Indeed, women with hypothalamic amenorrhea or oligomenorrhea experience premature bone loss, and delayed sex hormone production, as seen in constitutional delay of growth and puberty, is associated with reduced BMD [7, 14, 15]. Conversely, excessive or early exposure to estrogens, as observed in central precocious puberty (CPP), may accelerate skeletal maturation and influence the timing and pattern of peak bone mass acquisition [10-12].

In females, CPP is characterized by premature activation of the hypothalamic–pituitary–ovarian (HPO) axis, leading to early and sustained elevations in circulating sex steroids. In contrast, premature thelarche (PT), defined by early breast development without full HPO axis activation, is typically considered a benign variant with minimal impact on bone maturation [16].

Notably, previous studies have demonstrated that the increase in BMD during puberty is more pronounced at trabecular-rich sites such as the lumbar spine, compared to cortical bone sites such as the femoral shaft [2, 4-6]. This differential sensitivity reflects the distinct responses of bone compartments to hormonal stimuli during growth [4-6]. In our clinical experience, girls with central precocious or early puberty appear to exhibit disproportionately elevated BMD values at the lumbar spine relative to total body less head (TBLH) BMD. This observation led us to hypothesize that specific densitometric patterns may be associated with early pubertal activation. Given that body weight is also a known determinant of BMD [5, 6], we extended our analysis beyond bone density to include assessments of body composition (lean mass, fat mass, and fat distribution), anthropometry, and biochemical markers.

The aim of this study was to investigate whether girls with rapidly progressive CPP or early puberty (EP) show a distinct bone and body composition profile compared to girls with PT and a prepubertal control (PC) group, using dual-energy X-ray absorptiometry (DXA)-derived parameters. By including both skeletal and metabolic indices, we sought to clarify whether early hormonal activation and adiposity are associated with specific densitometric patterns that may support clinical evaluation and long-term risk stratification.

## Study design

Retrospective, single-center, observational cohort study.

## Materials and methods

We retrospectively assessed the health records of 184 girls aged ≥5 years, referred to a tertiary-level academic center (Pediatric Endocrine Unit) between May 2011 and May 2024 for suspected CPP or EP, based on breast development (Tanner stage ≥ 2) before the age of 9 years, as reported by caregivers, and who underwent DXA scanning.

A total of 108 girls were diagnosed with CPP or EP and were classified as CPP/EP. This group comprised both girls who exhibited breast development (Tanner stage ≥ 2) before 8 years of age, consistent with CPP, and those with onset between 8 and 9 years, consistent with EP [17]. The diagnosis was confirmed based on clinical signs and biochemical evidence of HPO axis activation, defined as basal serum luteinizing hormone (LH) > 1 U/L or peak LH > 5 U/L following a GnRH stimulation test, in association with at least one of the following criteria: bone age (BA) advanced by ≥1 year, or height (H) or delta height minus target height (ΔH – TH) ≥ + 2 SDS [16]. These combined features are consistent with the definition of rapidly progressive CPP or EP [18]. Girls in this group could present with or without pubarche.

Seventy-six girls were classified as premature thelarche (PT), defined as breast development (Tanner stage ≥ 2) before age 9 without biochemical evidence of central activation (basal LH < 0.3 U/L and/or peak LH < 5 U/L after the GnRH stimulation test), and without clinical signs of rapid pubertal progression. Girls in this group could also present with or without pubarche.

In accordance with routine clinical practice at our center, the GnRH stimulation test was performed when clinically indicated (eg, progressive pubertal signs, accelerated growth velocity, advanced BA, or basal gonadotropin levels suggestive of central activation). In patients with a clearly pubertal basal hormonal profile (LH > 1 U/L) together with rapid clinical progression (breast Tanner stage 3-4, height SDS ≥ 2 or height SDS – target height SDS ≥ 2, and bone age advanced by ≥1 year over chronological age [CA]), stimulation testing was not deemed necessary. In girls with premature breast development who did not show clinical or auxological features suggestive of rapidly progressive puberty and whose basal LH levels were not suggestive of central activation, a conservative approach with longitudinal clinical follow-up was commonly adopted, and GnRH testing was not systematically required.

Finally, 47 age-matched prepubertal girls without clinical signs of puberty (Tanner stage 1) who underwent DXA scans between April 2009 and June 2023 were included as prepubertal controls (PC). In these girls DXA was performed in the context of previous research protocols on bone development at our center. All girls with chronic, genetic, or metabolic diseases known to affect bone health were excluded, in order to ensure comparability with the study population.

Exclusion criteria included peripheral precocious puberty, genetic syndromes, skeletal dysplasia, brain tumors, neuromuscular diseases, extremely preterm birth (<28 weeks gestation), and absence of a DXA scan. We also excluded girls with known primary or secondary conditions (genetic, chronic, or iatrogenic) that may affect bone metabolism or contribute to skeletal fragility.

The study was approved by the local Ethical Committee (CER Liguria Register No.: 469/2022 - DB id 12639) and written informed consent was obtained from parents or caregiver of patients after full explanation of the study in accordance to the Declaration of Helsinki.

### Data collection

We collected the following data: anamnestic information (age at thelarche and pubarche, maternal age at menarche, family history of precocious puberty, gestational age at birth, birth weight SDS, and ethnicity, categorized as European or non-European); anthropometric and clinical data (weight—W, height—H SDS, body mass index—BMI SDS, target height—TH SDS, height minus target height difference [ΔH – TH SDS], and pubertal Tanner stages); biochemical parameters (basal and peak gonadotropins including follicle-stimulating hormone [FSH] and LH after GnRH stimulation test, 17-beta estradiol [E2], insulin-like growth factor 1 [IGF-1], and IGF-1 SDS); radiological assessments (BA, uterine length and transverse diameter, and ovarian volumes); and body composition parameters derived from whole-body DXA scans (Lunar Prodigy and Lunar iDXA systems).

A family history of precocious puberty was defined as menarche occurring before the age of 10.25 years in the patient's mother, sister, or grandmother [19].

### Clinical data

Pubertal maturation was assessed through breast inspection and palpation and classified according to Tanner stage [20]. Height (to the nearest 0.1 cm) and weight (to the nearest 0.1 kg) were measured using a calibrated Harpenden stadiometer and an electronic scale, respectively. TH was calculated according to the formula: (father's height + mother's height − 13 cm)/2 [21], by measuring parents' height with calibrated Harpenden stadiometer whenever possible. Standard deviation scores (SDS) for the children's height and TH were calculated according to Tanner Whitehouse (1976) reference charts [22]. The difference between the child's height SDS and TH SDS was expressed as height minus target height SDS (H − TH SDS).

BMI SDS was calculated by using the LMS method presented in the World Health Organization chart [23]. According to consensus position statement of the Italian Society for Pediatric Endocrinology and Diabetology and the Italian Society of Pediatrics [24], we defined overweight as BMI between 85°-97° percentile (p) and obesity as BMI > 97°p of the 2007 WHO charts [25].

### Biochemical data

FSH, LH, E2 were measured after venous fasting blood sampling between 08.00 and 09.00 Am with electrochemiluminescence method, by using a Roche Elecsys 2010 Immunoassay Analyzer (Roche Diagnostics).

The GnRH test was performed by administering i.v. Lutrelef™ (synthetic form of natural gonadotropin releasing hormone)—Ferring at the dose of 100 µg/m^2^ (maximum dose: 100 µg) iv. Blood samples were taken at 0, 15, 30, and 60 minutes for the determination of LH and FSH.

All serum IGF-1 samples were measured by chemiluminescent immunometric assay (Immulite 2000; Diagnostic Products Corporation). The intra and interassay coefficients of variation were 3.4% and 7.1%, respectively, and the sensitivity of the method was 2.6 nmol/L. IGF-1 SDS was calculated using reference data tables [26, 27].

Serum levels of adiponectin, C-terminal telopeptide of type I collagen (CTX), and bone-specific alkaline phosphatase (BAP) were measured as biomarkers of metabolic and bone turnover status and interpreted according to age- and sex-specific normative data [28-30].

Serum CrossLaps® (CTX-I) levels were determined using the ELISA kit (Immunodiagnostic Systems, IDS, Boldon, UK; RRID: AB_2923399), according to the manufacturer's instructions.

BAP was measured using the MicroVue™ BAP EIA kit (Quidel Corporation, San Diego, CA, USA; catalog no. 8012; RRID:AB_3095588), following the manufacturer's protocol.

All assays were performed on an automated DSX system (Technogenetics-Bouty, Milan, Italy). Intraassay coefficients of variation were <3% for CTX-1 and <4% for BAP, while interassay variability was <5% for both markers.

### Radiological data

BA was determined based on radiography of the left hand and wrist according to Greulich and Pyle [31], using the BoneXpert™ software [32] and by an experienced rater (D.F.). ΔBA − A was considered as the difference between BA and chronological age (A). Pelvic transabdominal ultrasonography (US) was performed with a curvilinear 2-7 MHz probe by two skilled gynecologists (R.A. and V.T.) working at our Centre. Uterine length, transverse uterine diameter, and the height, width, and length of the ovaries were measured; the volume of each ovary was calculated according to the ellipse formula (length × transverse diameter × fundal anteroposterior diameter × 0.5233).

### DXA-derived assessment of bone status and body composition parameters

All girls underwent total-body less head (TBLH) and lumbar spine (L1–L4) DXA examinations using a Lunar Prodigy Advance DXA system (software version 16; GE Medical Systems Lunar) until April 2020, and a Lunar iDXA system (software version 18; GE Medical Systems Lunar) thereafter. The DXA machine's calibration was checked daily with a GE Lunar block calibration phantom; the results were within the acceptable range of variation. Two different trained operators (N.D.I., A.A.) positioned the patients, performed all scans and manually checked the quality of the single scan and edited, as necessary, repositioning correctly the regions of interest (ROI). The ROI were automatically calculated by the enCORE software for total body, arms, legs, trunk, android, and gynoid regions.

The following bone-related DXA parameters were assessed: TBLH BMD (g/cm²) and corresponding *Z*-score, as well as lumbar spine L1-L4 BMD (g/cm²) and *Z*-score. Total BMC (g) was also recorded at both the TBLH and L1-L4 sites. To minimize the confounding effect of bone size on areal BMD, we calculated lumbar spine bone mineral apparent density (BMAD), a size-adjusted index of volumetric mineralization [33, 34]. Lumbar spine BMAD was calculated according to Crabtree et al [35]. Subsequently, BMAD values were standardized to age- and sex-specific reference data and expressed as *Z*-scores [35]. Additionally, the trabecular bone score (TBS) was evaluated in girls of groups CPP/EP and PT as an indirect measure of trabecular microarchitecture. TBS values were automatically calculated by the Lunar iDXA system from L1-L4 DXA scans using dedicated software, based on the assessment of gray-level texture variations in 2D lumbar spine images.

DXA-derived body composition data included total fat mass (FM, kg and %), total fat-free mass (FFM, kg), truncal fat mass (kg and %), upper limb lean mass (kg), and lower limb lean mass (kg). The GE Lunar Prodigy DXA system automatically delineates android and gynoid regions using the enCORE software based on standardized anatomical landmarks. The android region was defined with its lower boundary at the top of the pelvic horizontal line and its upper boundary set at 20% of the distance between the pelvis and femoral neck; lateral boundaries were defined by the inner arm contours in the standard supine scan position [36]. The gynoid region was delineated inferior to the android region, with its upper boundary located 1.5 times the android height below the pelvic line and its lower boundary 2 times the android height below this point. Lateral boundaries were defined by the outer thigh contours [36]. The android to gynoid fat (A/G) ratio was calculated using the fat mass values (kg) within these two predefined regions of interest.

From these measurements, the following body composition indices were calculated: appendicular lean mass index (ALMI = [upper + lower limb lean mass]/height², kg/m²), fat mass index (FMI = FM/height², kg/m²), and fat-free mass index (FFMI = FFM/height², kg/m²) [37]. Fat-free mass (FFM) refers to the total mass excluding all fat tissue. It is calculated by subtracting fat mass from total body weight (FFM = total body weight − fat mass).

### Statistical analysis

The distribution of continuous variables was assessed using the Kolmogorov–Smirnov test. Descriptive statistics were used to present the sample: continuous variables were described using the mean and standard deviation (SD), while categorical variables were presented as absolute values and percentages.

The sample was divided into three groups based on inclusion criteria. Variables in each group were compared: continuous variables were analyzed using the nonparametric Mann–Whitney U test. Categorical variables were compared using Pearson's *χ*² test or Fisher's exact test.

The software used for the analysis was the Statistical Package for the Social Sciences for Windows, version 22 (SPSS Inc., Armonk, NY). The threshold for statistical significance was set at a two-tailed *P*-value of less than .05.

## Results

**Figure 1:**
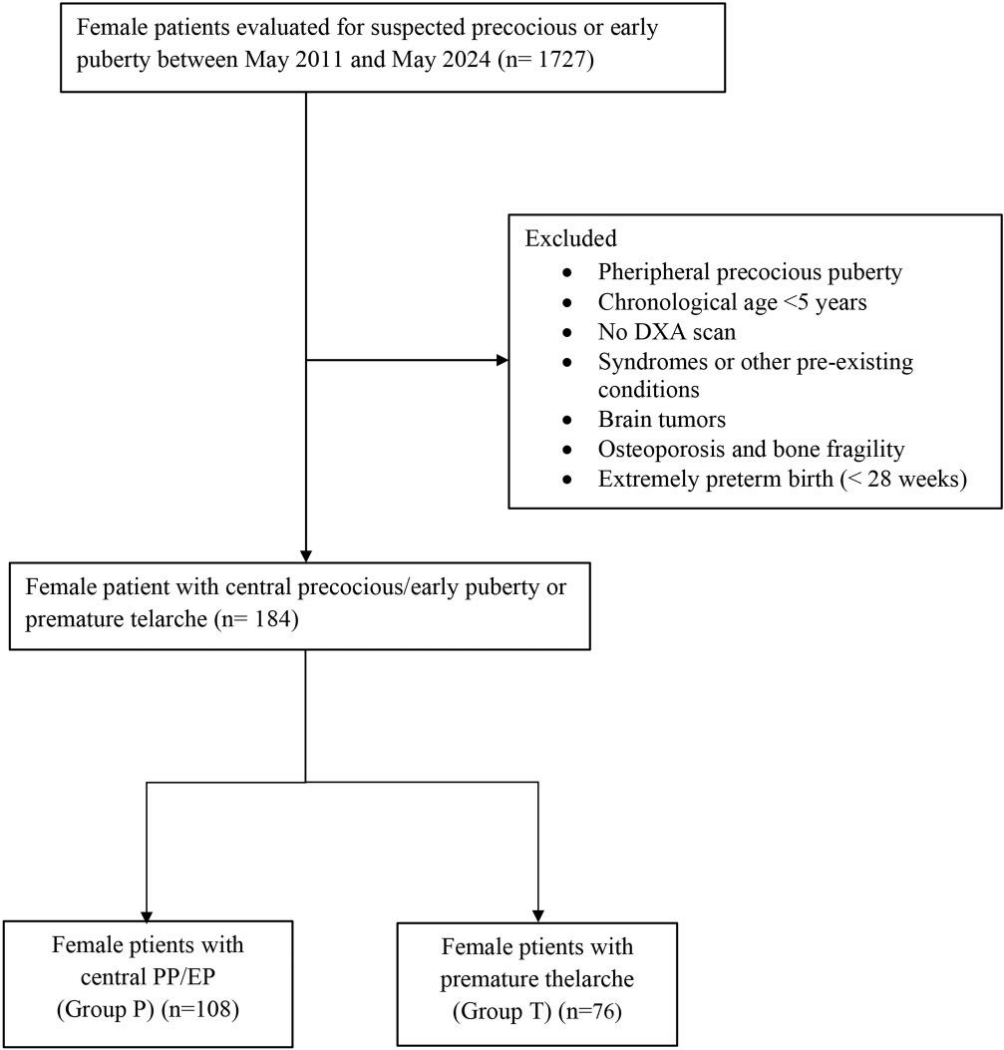
Flowchart illustrating the selection of 184 girls from 1727 evaluated for suspected early puberty. After applying exclusion criteria, 108 were classified with central precocious/early puberty (CPP/EP) and 76 with premature thelarche (PT).

A total of 1727 girls with breast development before the age of 9 years were evaluated for suspected precocious or EP between May 2011 and May 2024. Based on inclusion and exclusion criteria, we analyzed the clinical, biochemical, ultrasound, and DXA-derived data from 184 girls, including108 classified as CPP/EP and 76 classified as PT (Fig. 1). In addition, 47 age-matched prepubertal girls without clinical signs of puberty (Tanner stage 1) who underwent DXA scans between April 2009 and June 2023 were included as PC.

### Central precocious puberty/early puberty

In 92 of 108 girls (85.2%) with CPP/EP, breast development started before the age of 8 years, whereas in 16 (14.8%) it occurred between 8 and 9 years. In 7 girls (7.6%), pubarche preceded thelarche. A family history for PP was reported in 20 girls (18.5%). At presentation, breast development was most frequently Tanner stage 2 (34.3%) or stage 3 (53.7%), while stage 4 accounted for 12.0%. Pubic hair development was mainly Tanner stages 1 (38.0%) and 2 (41.7%), whereas stages 3 and 4 were less frequent (18.5% and 1.9%, respectively).

A GnRH stimulation test was performed in 82 patients (75.9%), confirming HPO axis activation. The remaining 26 patients were classified without stimulation test because they had clearly pubertal basal LH (>1 U/L) together with markers of rapid progression, including breast Tanner stages 3 to 4, height SDS or height SDS − target height SDS ≥2, and BA advanced by ≥1 year over chronological age.

### Premature thelarche

In 69 of 76 girls (90.8%) with PT, breast development started before the age of 8 years, whereas in 7 (9.2%) it occurred between 8 and 9 years. Pubarche preceded thelarche in 16/69 girls (23.2%) with thelarche onset before 8 years and in 3/7 girls (42.9%) with thelarche onset between 8 and 9 years, corresponding to 19/76 (25.0%) of the PT cohort. A family history of precocious puberty was reported in 13 girls (17.1%).

At presentation, breast development was most frequently Tanner stage 2 (63.1%), followed by stage 3 (34.2%), whereas stage 4 was rare (2.6%). Pubic hair development was predominantly Tanner stage 1 (48.7%) or 2 (31.6%); stage 3 was observed in 19.7% and stage 4 was virtually absent. A GnRH stimulation test was performed in 55 patients, excluding HPO axis activation. The remaining 21 girls did not undergo stimulation testing because they lacked clinical and auxological features suggestive of rapidly progressive central puberty.

### Prepubertal controls

All PC girls were Tanner stage 1 at the clinical visit. A family history for precocious puberty was reported in 1 girl (2.1%).

### Weight categories and ethnic distribution of the study population

In our cohort, 19.5% of girls (*n* = 45/231) were classified as having obesity. The prevalence of obesity was 15.7% (*n* = 17/108) in CPP/EP, 27.6% (*n* = 21/76) in PT, and 14.9% (*n* = 7/47) in PC.

Among the 26 girls whose first pubertal sign was pubarche, obesity was present in 50.0% of cases (*n* = 13/26); of these, 84.6% belonged to the PT group.

Overall, 19.5% (*n* = 45/231) had overweight, including 23.2% (*n* = 25/108) in CPP/EP, 21.1% (*n* = 16/76) in PT, and 8.5% (*n* = 4/47) in PC. Overweight was also observed in 15.4% (*n* = 4/26) of girls with pubertal onset with pubarche.

The majority of the cohort, 61.0% (*n* = 141/231), had normal weight, including 61.1% (*n* = 66/108) in CPP/EP, 51.3% (*n* = 39/76) in PT, and 76.6% (*n* = 36/47) in PC.

Across breast Tanner stages, the percentage of girls with overweight/obesity increased with pubertal progression in CPP/EP (29.7% at stage 2, 41.4% at stage 3, and 53.8% at stage 4) whereas it remained consistently high in PT (45.8% at stage 2, 53.8% at stage 3, and 50% at stage 4).

In our cohort, 14.7% (*n* = 34/231) of the girls were of non-European origin; among them, 32.3% (11/34) were of Honduran descent. Non-European origin accounted for 23.1% (25/108) of girls in CPP/EP and 11.8% (9/76) in PT. Five girls (2.16%) were adopted, all belonging to the CPP/EP group.

### Comparative analysis between CPP/EP and PC

Compared with PC, girls with CPP/EP were significantly taller and showed higher weight and BMI SDS (Table 1; Fig. S1A [38]). They also had a more advanced BA and a greater bone age − chronological age difference (ΔBA − CA), although BA assessment was performed at a significantly older chronological age than in PC.

**Table 1:** Clinical, biochemical and densitometric characteristics in girls with CPP/EP, PT, and PC

Parameter	Group P (*n* = 108)	Group T (*n* = 76)	Group C (*n* = 47)	P vs C *P*-value	P vs T *P*-value	T vs C *P*-value
*Clinical characteristics*		*Mean ± SD*				
Age at visit (years)	8.02 ± 0.82	7.70 ± 1.05	7.66 ± 1.37	.69	.05	.69
Age of mother's menarche (years)	11.64 ± 1.71	12.05 ± 1.74	12.66 ± 1.72	.**003**	.10	.08
Gestational age at birth (weeks)	39.07 ± 2.08	39.08 ± 1.82	38.33 ± 2.97	.39	.67	.56
Birth weight SDS	0.03 ± 1.15	−0.03 ± 1.05	−0.17 ± 1.00	.39	.77	.64
Height SDS	1.41 ± 1.13	1.25 ± 1.04	−0.13 ± 1.33	**<**.**001**	.20	**<**.**001**
Weight (kg)	31.633 ± 6.636	31.416 ± 8.579	25.613 ± 7.941	**<**.**001**	.55	**<**.**001**
BMI SDS	0.84 ± 1.03	1.03 ± 1.18	0.36 ± 1.42	.**03**	.31	.**01**
Target height SDS	−0.29 ± 0.79	0.01 ± 0.97	−0.12 ± 0.83	.30	.06	.59
Delta H-TH SDS	1.72 ± 1.18	1.23 ± 1.04	−0.01 ± 1.24	**<**.**001**	.**005**	**<**.**001**
Age at BA evaluation (A) (years)	7.86 ± 0.88	7.56 ± 1.03	7.21 ± 1.32	.**02**	.05	.24
BA (years)	9.51 ± 1.29	8.78 ± 1.44	6.68 ± 1.32	**<**.**001**	**<**.**001**	**<**.**001**
Delta BA—A(years)	1.48 ± 1.00	1.07 ± 1.12	−0.56 ± 1.06	**<**.**001**	.**031**	**<**.**001**
						
*Endocrine data*						
Baseline LH (U/L)	1.63 ± 2.00	0.07 ± 0.16	0.04 ± 0.09	**<**.**001**	**<**.**001**	.68
Baseline FSH (U/L)	4.48 ± 2.58	1.93 ± 1.46	1.78 ± 1.01	**<**.**001**	**<**.**001**	.63
Peak LH (U/L)	14.01 ± 14.78	3.08 ± 1.19	NA	NA	**<**.**001**	NA
Peak FSH (U/L)	13.99 ± 6.98	10.13 ± 2.92	NA	NA	**<**.**001**	NA
Basal LH-FSH ratio	0.37 ± 0.56	0.04 ± 0.08	0.02 ± 0.03	**<**.**001**	**<**.**001**	.89
Peak LH-FSH ratio	1.19 ± 1.09	0.31 ± 0.13	NA	NA	**<**.**001**	NA
E2 (pg/mL)	24.54 ± 23.25	5.67 ± 9.75	5.91 ± 11.76	**<**.**001**	**<**.**001**	.38
						
*Biochemical data*						
Adiponectin (mg/L)	9.14 ± 4.17	9.27 ± 5.87	12.77 ± 5.86	.**007**	.79	.**005**
BAP (µg/L)	97.79 ± 35.83	97.77 ± 32.80	104.56 ± 30.22	.22	.82	.39
CTX (ng/mL)	2.05 ± 0.82	1.71 ± 0.65	1.89 ± 0.62	.64	.16	.24
IGF-1 SDS	2.62 ± 1.37	1.23 ± 1.09	−0.13 ± 0.98	**<**.**001**	**<**.**001**	**<**.**001**
IGF-1 SDS adjusted for BA	1.52 ± 0.96	0.70 ± 0.91	−0.09 ± 0.91	**<**.**001**	**<**.**001**	**<**.**001**
*Densitometric data*						
Android fat mass (kg)	0.531 ± 0.355	0.657 ± 0.473	0.434 ± 0.372	.**01**	.29	.**006**
Gynoid fat mass (kg)	1.738 ± 0.632	1.831 ± 0.825	1.359 ± 0.699	**<**.**001**	.82	**<**.**001**
Android–gynoid fat ratio	0.282 ± 0.093	0.320 ± 0.112	0.287 ± 0.101	.83	.**02**	.10
Total fat mass (kg)	10.155 ± 3.969	10.897 ± 4.956	8.172 ± 4.385	**<**.**001**	.65	**<**.**001**
Total fat-free mass (kg)	21.672 ± 3.471	20.603 ± 3.899	17.546 ± 4.012	**<**.**001**	.**03**	**<**.**001**
Body fat %	33.27 ± 7.26	35.64 ± 8.29	32.76 ± 7.60	.43	.07	.05
Truncal fat %	28.23 ± 8.84	31.19 ± 10.63	27.10 ± 9.94	.34	.08	.**03**
FMI (kg/m^2^)	5.66 ± 2.02	6.27 ± 2.42	5.33 ± 2.39	.12	.18	.**02**
Upper limbs lean mass (kg)	1.906 ± 0.373	1.806 ± 0.426	1.475 ± 0.443	**<**.**001**	.06	**<**.**001**
Lower limbs lean mass (kg)	6.770 ± 1.376	6.362 ± 1.613	5.170 ± 1.601	**<**.**001**	.**04**	**<**.**001**
ALMI (kg/m^2^)	4.85 ± 0.58	4.75 ± 0.68	4.35 ± 0.79	**<**.**001**	.22	.**01**
FFMI (kg/m^2^)	12.15 ± 1.05	12.04 ± 1.05	11.61 ± 1.24	.**005**	.30	.05
BMD TBLH *Z*-score	−0.04 ± 0.95	0.16 ± 0.92	−0.33 ± 1.29	.09	.20	.**01**
BMD L1-L4 *Z*-score	0.56 ± 0.82	0.59 ± 0.95	−0.09 ± 1.35	**<**.**001**	.50	.**002**
Delta BMD L1-L4—TBLH *Z*-score	0.61 ± 0.66	0.44 ± 0.62	0.23 ± 1.04	.07	.16	.16
BMC TBLH (g)	793.3 ± 170.4	767.8 ± 186.6	632.9 ± 192.6	**<**.**001**	.53	**<**.**001**
BMC L1-L4 (g)	22.8 ± 4.9	22.6 ± 5.5	18.8 ± 5.9	**<**.**001**	.23	**<**.**001**
BMAD L1-L4 *Z*-score	−0.12 ± 0.83	−0.03 ± 0.94	−0.20 ± 1.07	.30	.71	.35
L1-L4 TBS*^a^*	1.29 ± 0.67	1.32 ± 0.68	NA	NApp	.**03**	NApp
						
*Ultrasound data*						
Uterus length (mm)	35.81 ± 8.28	31.73 ± 9.42	NA	NA	**<**.**001**	NA
Uterus anteroposterior diameter (mm)	10.85 ± 4.29	9.84 ± 8.04	NA	NA	.**001**	NA
Right ovary volume (cm³)	1.85 ± 1.33	1.74 ± 1.38	NA	NA	.52	NA
Left ovary volume (cm³)	1.73 ± 1.65	1.58 ± 1.43	NA	NA	.68	NA

Bold values indicate statistically significant differences (*P* < .05).Abbreviations: ALMI, appendicular lean mass index; BA, bone age; BAP, bone alkaline phosphatase; BMAD, bone mineral apparent density; BMC, bone mineral content; BMD, bone mineral density; CTX, C-terminal telopeptide of type I collagen; delta BA, delta age-bone age; E2, estradiol; FFMI, fat-free mass index; FMI, fat mass index; H, height; IGF-1, insulin-like growth factor 1; TBLH, total body less head, L1-L4, lumbar spine; TBS, trabecular bone score; TH, target height.

^*a*^Missing TBS: 13 (P), 27 (T).

Consistent with activation of the HPO axis, baseline LH, FSH, and estradiol levels were significantly elevated in CPP/EP.

Compared with PC, girls with CPP/EP showed significantly lower adiponectin levels and higher IGF-1 SDS values, even after adjustment for BA.

Differences in body composition between groups were subsequently examined (Fig. S1B-S1D [38]). Girls with CPP/EP showed greater total fat mass than PC. Despite these differences in absolute fat mass, no significant differences were observed in FMI or fat distribution, as total body fat percentage and trunk fat percentage did not differ between the two groups. Additionally, girls with CPP/EP exhibited significantly higher lean mass, as well as elevated ALMI and FFMI. BMC was higher in CPP/EP at both the lumbar spine and total body, whereas only lumbar spine BMD *Z*-scores were significantly higher. The difference between lumbar spine and TBLH BMD *Z*-score showed a nonsignificant trend toward higher values in CPP/EP compared with PC (*P* = .07). Lumbar spine BMAD *Z*-scores did not differ between CPP/EP and PC.

### Comparative analysis between CPP/EP and PT

Significant differences were observed between CPP/EP and PT across several parameters (Table 1). Girls with CPP/EP had significantly higher height SDS − target height SDS, BA, and BA − CA difference, as well as higher estradiol levels and both basal and stimulated LH and FSH levels at the GnRH stimulation test. IGF-1 SDS values were significantly higher in CPP/EP, even after adjustment for BA.

IGF-1 SDS values were significantly higher in CPP/EP, even after adjustment for BA.

Uterine longitudinal and anteroposterior diameters were significantly greater in CPP/EP than in PT. whereas ovarian volume did not differ significantly between the two groups.

Girls with PT had significantly higher A/G ratio (Fig. S1B [38]). In contrast, girls with CPP/EP had significantly higher total FFM and lower limbs lean mass than those with PT.

Finally, L1-L4 TBS was higher in PT compared with CPP/EP (*P* = .03) (Fig. S2 [38]). L1-L4 BMAD *Z*-scores did not differ significantly between CPP/EP and PT.

### Comparative analysis between PT and PC

Compared with PC, girls with PT were taller, even relative to their target height, and had a higher weight and BMI SDS (Table 1; Fig. S1A [38]). They also had a more advanced BA and a greater bone age-chronological age difference. No differences were observed between PT and PC in basal or stimulated gonadotropin levels, nor in estradiol concentrations.

Adiponectin levels were significantly higher in PC than in PT.

IGF-1 SDS values were significantly higher in PT, even after adjustment for BA.

Regarding body composition parameters, girls with PT showed higher overall adiposity and lean mass, including significantly higher android and gynoid fat mass, total and FFM, trunk fat percentage, and arms and legs lean mass, as well as higher FMI and ALMI, with a trend toward higher FFMI values (*P* = .05) compared with controls (Fig. S1B-S1D [38]).

BMC and BMD *Z*-scores SDS, both in the spine and the whole body, such as L1-L4 TBS, were higher in PT than in PC. L1-L4 BMAD *Z*-scores did not differ significantly between the PT and PC.

### Comparative analysis of PT girls with thelarche and pubarche as first sign of puberty

Within PT, significant differences were observed in BMI SDS (Fig. S3A [38]), BA and body composition between girls whose first pubertal sign was thelarche and those whose first sign was pubarche (Table 2). Girls presenting with pubarche at pubertal onset exhibited significantly greater H SDS, weight, and BMI SDS, as well as a significantly more advanced bone age. These girls had also significantly higher CTX values.

**Table 2:** Clinical, biochemical and densitometric findings in girls with thelarche or pubarche as the first pubertal sign in the PT group

	Girls with thelarche as first sign of puberty *N* = 57	Girls with pubarche as first sign of puberty *N* = 19	*P*-value
*Clinical characteristics*	*Mean ± SD*		
Age at visit (years)	7.65 ± 1.09	8.02 ± 0.56	.41
Age at thelarche (years)	6.53 ± 1.82	7.21 ± 0.92	.31
Age of mother's menarche (years)	12.0 ± 1.55	12.2 ± 1.48	.49
Gestational age at birth (weeks)	39.28 ± 1.55	38.26 ± 2.53	.12
Birth weight SDS	−0.07 ± 0.99	0.14 ± 1.31	.90
Height SDS	1.07 ± 0.98	1.77 ± 1.08	.**04**
Weight (kg)	29.261 ± 7.928	37.941 ± 7.528	**<**.**001**
BMI SDS	0.74 ± 1.12	1.75 ± 1.04	.**002**
Target height SDS	−0.01 ± 1.01	0.06 ± 0.94	.53
Delta H-TH SDS	1.06 ± 1.04	1.71 ± 0.99	.05
Age at BA evaluation (A) (years)	7.51 ± 1.05	7.82 ± 0.68	.47
Bone age (BA)	8.58 ± 1.44	9.39 ± 1.06	.**03**
Delta BA-A (years)	0.93 ± 1.14	1.36 ± 0.99	.16
			
*Endocrine data*			
Baseline LH (U/L)	0.08 ± 0.16	0.08 ± 0.18	.83
LH peak (U/L)	3.19 ± 1.21	2.75 ± 1.22	.20
Baseline FSH (U/L)	2.04 ± 1.57	1.71 ± 1.16	.30
FSH peak (U/L)	10.31 ± 2.98	9.30 ± 2.85	.18
basal LH-FSH ratio	0.04 ± 0.08	0.04 ± 0.08	.99
Peak LH-FSH ratio	0.31 ± 0.13	0.32 ± 0.18	.45
E2 (pg/mL)	5.96 ± 10.93	3.88 ± 5.39	.97
			
*Biochemical data*			
Adiponectin (mg/L)	10.54 ± 6.93	7.33 ± 1.43	.13
BAP (µg/L)	97.15 ± 36.77	97.39 ± 23.26	.93
CTX (ng/mL)	1.55 ± 0.66	2.13 ± 0.49	.**03**
IGF-1 SDS	1.17 ± 1.04	1.20 ± 1.06	.94
IGF-1 SDS adjusted for BA	0.70 ± 0.87	0.54 ± 0.76	.70
			
*Ultrasound data*			
Uterus length (mm)	31.75 ± 10.15	30.81 ± 6.72	.99
Uterus anteroposterior diameter (mm)	10.38 ± 8.84	7.73 ± 4.24	.11
Right ovary (cm^3^)	1.81 ± 1.46	1.23 ± 0.91	.24
Left ovary (cm^3^)	1.48 ± 1.09	2.03 ± 2.42	.64
			
*Densitometric data*			
Android fat mass (kg)	0.547 ± 0.438	0.992 ± 0.458	**<**.**001**
Gynoid fat mass (kg)	1.605 ± 0.735	2.496 ± 0.750	**<**.**001**
A/G ratio	0.301 ± 0.110	0.378 ± 0.109	.**02**
Total fat mass (kg)	9.573 ± 4.400	14.793 ± 4.696	**<**.**001**
Total fat-free mass (kg)	17.300 ± 3.773	19.819 ± 2.869	.**006**
Body fat %	33.51 ± 7.94	41.60 ± 6.49	**<**.**001**
Truncal fat %	28.68 ± 10.18	38.37 ± 9.12	**<**.**001**
FMI (kg/m^2^)	11.90 ± 1.00	12.35 ± 1.16	**<**.**001**
Upper limbs lean mass (kg)	1.738 ± 0.436	2.011 ± 0.327	.**006**
Lower limbs lean mass (kg)	6.054 ± 1.626	7.320 ± 1.213	.**002**
ALMI (kg/m^2^)	4.62 ± 0.67	5.07 ± 0.60	.**01**
FFMI (kg/m^2^)	11.90 ± 1.00	12.35 ± 1.16	.08
BMD TBLH *Z*-score	−0.01 ± 0.84	0.55 ± 1.09	.11
BMD L1-L4 *Z*-score	0.44 ± 0.93	1.08 ± 0.91	.**02**
BMAD L1-L4 *Z*-score	−0.20 ± 0.91	0.45 ± 0.88	.**01**
Delta BMD L1-L4—TBLH *Z* score	0.43 ± 0.62	0.56 ± 0.58	.45
BMC TBLH (g)	736.1 ± 184.4	876.1 ± 151.8	.**002**
BMC L1-L4 (g)	22.9 ± 5.8	21.9 ± 4.7	.70
L1-L4 TBS*^a^*	1.31 ± 0.07	1.33 ± 0.08	.65

Bold values indicate statistically significant differences (*P* < .05).Abbreviations: ALMI, appendicular lean mass index; BA, bone age; BAP, bone alkaline phosphatase; BMAD, Bone Mineral Apparent Density; BMC, bone mineral content; BMD, bone mineral density; CTX, C-terminal telopeptide of type I collagen; delta BA, delta age-bone age; E2, estradiol; FFMI, fat-free mass index; FMI, fat mass index; H, height; IGF-1, insulin-like growth factor 1; TBLH, total body less head; L1-L4, lumbar spine; TBS, trabecular bone score; TH, target height.

^*a*^Missing TBS: 22 (Thelarche), 5 (Pubarche).

Girls with pubarche as the initial sign of puberty exhibited significantly greater adipose tissue, both in absolute values and percentages, along with significantly higher A/G distribution and a significantly increased A/G ratio (Fig. S3B [38]). In these girls, FFM, total body, and truncal fat percentage, as well as lean mass in the upper and lower limbs, were significantly higher, with a trend toward higher FFMI values (*P* = .08) (Fig. S3C [38]) and notably elevated FMI (Fig. S3D [38]) and ALMI. Similarly, they exhibited a higher lumbar spine BMD *Z*-score and a higher BMC in the whole body.

### Comparative analysis of girls with overweight/obesity and normal weight in CPP/EP, PT, and PC

In CPP/EP, 67 girls (62.0%) had overweight/obesity and 41 (38.0%) were normal-weight (Table S1 [38]). Girls with overweight/obesity were significantly taller (*P* = .02), with only a trend toward a higher height SDS − target height SDS (*P* = .05); they were also heavier (*P* < .001) and had a higher BMI SDS (*P* < .001). A trend toward more advanced BA and greater BA − CA difference was also observed (*P* = .05). No significant hormonal differences were detected, except for peak FSH, which was significantly higher in normal-weight girls (*P* = .002). Regarding body composition, girls with overweight/obesity had significantly greater absolute and percentage fat mass (*P* < .001) at total body, trunk, android, and gynoid sites, along with a higher A/G ratio, but also greater lean mass and significantly higher FMI, ALMI, and FFMI. BMD *Z*-scores were significantly higher (*P* < .001) both at the lumbar spine and at TBLH, where BMC was also greater (*P* < .001). Importantly, L1-L4 BMAD *Z*-scores were significantly higher in girls with overweight/obesity compared to their normal-weight peers (*P* < .001).

In PT, 39 girls (51.3%) had overweight/obesity and 37 (48.7%) were normal-weight (Table S2 [38]). Girls with overweight/obesity were significantly taller (*P* < .001), including relative to TH SDS (*P* < .001); they were also heavier (*P* < .001) and had a higher BMI SDS (*P* < .001). They presented with more advanced bone age and greater BA − CA difference (*P* < .001). Hormonal assessment revealed higher basal LH and LH peak values in normal-weight girls (*P* = .005) and significantly higher basal FSH (*P* = .02). Body composition showed that girls with overweight/obesity had significantly greater absolute and percentage fat mass (*P* < .001) at total body, trunk, android, and gynoid sites, with a higher A/G ratio, but also greater lean mass and higher FMI, ALMI, and FFMI. BMD *Z*-scores were significantly higher (*P* < .001) at both lumbar spine and TBLH, where BMC was also greater (*P* < .001). In addition, the ΔBMD *Z*-score L1-L4—TBLH was significantly greater in girls with overweight/obesity (*P* = .04). Consistently, L1-L4 BMAD *Z*-scores were significantly higher in girls with overweight/obesity compared to those with normal weight (*P* = .007).

In PC, 36 girls (76.6%) had overweight/obesity and 11 (23.4%) were normal-weight (Table S3 [38]). Girls with overweight/obesity were significantly taller (*P* = .008), including relative to TH SDS (*P* = .003); they were also heavier (*P* < .001) and had a higher BMI SDS (*P* < .001). They also exhibited a significantly greater BA − CA difference (*P* = .01). No hormonal differences were observed between subgroups. Body composition analysis showed significantly greater absolute and percentage fat mass (*P* < .001) in girls with overweight/obesity at total body, trunk, android, and gynoid sites, without differences in the A/G ratio. They also displayed greater lean mass in both upper (*P* = .002) and lower limbs (*P* < .001), along with higher FMI, ALMI, and FFMI. BMD *Z*-scores were significantly higher at both lumbar spine (*P* = .003) and TBLH (*P* < .001), and BMC was significantly greater at both sites (*P* = .01 at lumbar spine and *P* = .004 at TBLH). Among controls, girls with overweight/obesity also showed significantly higher L1-L4 BMAD *Z*-scores, compared to their normal-weight peers (*P* = .01).

## Discussion

Our study provides a comprehensive assessment of bone and body composition, in girls with CPP/EP compared to those with PT and PC.

Body composition findings may indirectly reflect the potentially distinct hormonal influences involved in the initial phases of pubertal onset.

Our findings confirm that girls with CPP/EP had higher TBLH BMC, lumbar spine BMC, and lumbar spine BMD *Z*-scores than PCs, in line with previous studies [5, 39, 40]. However, BMD is strongly influenced by body and bone size in growing children; therefore, these differences should be interpreted primarily in the context of the greater stature and skeletal dimensions observed in CPP/EP compared with controls, rather than as evidence of intrinsically higher bone tissue density. In keeping with this interpretation, when lumbar spine BMAD (a size-adjusted estimate of volumetric density) was considered, no differences emerged among CPP/EP, PT, and controls, indicating no true volumetric increase in vertebral bone density across groups. CPP/EP girls were taller than controls and showed a tendency toward relatively higher lumbar spine vs TBLH BMD *Z*-scores; while this pattern could be compatible with site-specific hormonal effects at trabecular-rich regions [41], the absence of BMAD differences suggests that the between-group signal largely reflects skeletal size and maturation-related body proportions rather than changes in volumetric density.

Girls with PT also exhibited significantly higher BMC and BMD *Z*-scores than controls, despite lacking full HPO axis activation; importantly, these values were not significantly different from those of CPP/EP. When lumbar spine BMAD was used to account for bone size, no differences emerged among CPP/EP, PT, and controls, supporting the interpretation that the higher lumbar spine BMD *Z*-scores in CPP/EP and PT were primarily driven by skeletal size rather than by true volumetric increases in bone density. It is worth highlighting that PC girls were shorter and lighter than those with CPP/EP and PT, and these differences in body and skeletal size likely contributed to their lower BMD *Z*-scores.

Overall, bone mass accrual during EP is multifactorial, and while contributors such as hormonal and adiposity-related signals, as well as genetic and lifestyle factors, are known to play a role [42, 43], in our cohort these influences did not translate into BMAD differences across the study groups. The impact of adiposity was further clarified by BMI-stratified analyses. Consistent with evidence from a recent meta-analysis showing higher BMD in children with overweight/obesity [44], girls with CPP/EP and PT were heavier and had higher BMI than controls. This likely contributed to the greater BMC and BMD *Z*-scores observed, particularly in PT, where obesity was more prevalent and adiponectin levels were significantly lower, in line with its inverse relationship with adiposity [29]. When stratified by BMI categories, girls with overweight/obesity showed higher lumbar spine and TBLH BMD *Z*-scores across all groups, together with higher lumbar spine BMAD *Z*-scores, supporting the influence of excess weight on trabecular bone accrual, as previously reported in pediatric cohorts [45].

Beyond its impact on bone, increased adiposity also appears to influence pubertal progression, as reflected in the higher frequency of pubarche as the initial pubertal sign in PT. Consistent with previous reports, girls with pubarche tend to have higher BMI SDS than those with thelarche and than prepubertal peers [46]. Premature adrenarche, often presenting with early pubarche, is associated with accelerated growth and skeletal maturation, likely influenced by excess adiposity [47]. In line with this, Sopher et al showed that girls with premature adrenarche exhibit higher BMD and BMC than controls [48]. In our cohort, lumbar spine BMD *Z*-scores were highest in girls whose pubertal onset was marked by pubarche, supporting the hypothesis that adiposity and adrenal-derived androgens, via anabolic effects and peripheral aromatization [49, 50] may enhance mineralization at trabecular-rich sites. This was further confirmed by significantly higher lumbar spine BMAD values in these girls compared with those with thelarche onset, indicating a true volumetric gain in vertebral density. Girls with PT also exhibited higher TBS than those with CPP/EP; however, no TBS differences were observed within the PT group between pubarche-first and thelarche-first onset. TBS, an indirect marker of trabecular microarchitecture and an independent fracture-risk indicator in adults [51, 52], remains poorly characterized in pediatric populations [53]. In our study, TBS could be calculated in 88% of girls with CPP/EP, 64.5% of those with PT, and in only 3 controls, precluding statistical comparisons with prepubertal girls. The lower TBS in CPP/EP compared to PT may reflect the combined influence of adiposity, adrenal or gonadal hormones, or the rapid skeletal expansion typical of CPP/EP, where greater bone size may not correspond to proportional gains in trabecular structure [54], although alternative mechanisms cannot be excluded.

Regarding IGF-1 regulation, IGF-1 SDS was higher in both the CPP/EP and PT groups compared to controls, with the highest values in girls with CPP/EP, likely reflecting the combined effects of early HPO axis activation and adiposity. These findings are consistent with previous reports showing elevated IGF-1 in CPP/EP [55], and in children with obesity, in whom increased IGF-1 is linked to accelerated growth and skeletal maturation [56]. In our cohort, IGF-1 SDS did not differ between girls with overweight/obesity and those with normal weight within any group. Although prior studies have reported higher IGF-1 in girls with premature pubarche [57], no IGF-1 differences were observed within the PT group between those with pubarche-first and thelarche-first onset. Overall, these results suggest that factors beyond adiposity and potential adrenal androgen activity may contribute to IGF-1 variability in this population.

From a body composition perspective, both girls with CPP and those with PT showed greater absolute fat mass compared to prepubertal controls. However, FMI was significantly higher only in girls with PT, while no differences in FMI were observed between CPP/EP and controls. Total body fat percentage was similar across groups but tended to be higher in PT, who also showed increased trunk fat compared with controls and a tendency toward higher levels than CPP/EP. These patterns indicate greater overall and central adiposity in PT girls, particularly in those with pubarchal onset, consistent with their significantly higher A/G ratio compared with CPP/EP.

Both CPP/EP and PT girls showed higher total and appendicular lean mass, as reflected by increased ALMI values. However, only girls with CPP/EP exhibited significantly higher FFMI, indicating a more pronounced overall lean mass development consistent with their more advanced biological maturation. This aligns with prior evidence showing substantial gains in lean mass across pubertal progression, accompanied by parallel gains in fat mass [58]. The selective rise in FFMI among CPP/EP girls may relate to early activation of the hypothalamic–pituitary–gonadal axis, which enhances both fat and lean tissue accrual through estrogen and GH/IGF-1 activity. In contrast, although PT girls displayed increased lean mass, their FFMI remained only slightly above that of controls, consistent with the absence of biochemical signs of central activation. Importantly, these differences cannot be attributed to hormone levels alone, as body composition outcomes arise from a multifactorial interplay involving endocrine activity, adiposity-related signals, and broader genetic and environmental influences.

When the analysis was stratified by weight categories, FMI, ALMI, and FFMI were significantly higher in girls with overweight or obesity across all groups, confirming that excess weight amplifies both fat and lean compartments. The A/G ratio was also higher in overweight/obese girls in all groups, regardless of the type of pubertal activation.

Within the PT group, girls with pubarche as their initial pubertal sign had markedly higher regional and total fat and lean mass than those who entered puberty with thelarche. They also showed higher percentages of trunk fat, suggesting more pronounced and centralized adipose accumulation. In line with these observations, Kaya et al reported higher BMI and waist circumference SDS in girls with premature pubarche compared to healthy controls [59]. In this subgroup, FMI was also higher, further confirming increased adiposity. This is consistent with previous studies showing that girls with premature adrenarche tend to have greater fat mass [47, 60], and lean mass compared to their peers [61]. Indeed, girls with premature pubarche exhibited higher ALMI compared to those with thelarche onset, though FFMI did not differ significantly. This pattern suggests a preferential increase in appendicular muscle mass, potentially influenced by adrenal androgen activity, which may modestly enhance the anabolic effects of GH and IGF-1, particularly during puberty [62]. These observations may also reflect the contribution of other factors, indicating that multiple interacting mechanisms are likely involved. Recent evidence, summarized by Soriano-Guillén et al [63]. suggests that the organism's energy status acts as a metabolic switch for pubertal activation. Leptin indirectly stimulates Kiss1 neurons through hypothalamic pathways integrating metabolic cues, while mTOR, AMPK, and SIRT1 signaling modulate Kiss1 expression according to energy balance. In early-onset obesity, activation of mTOR and hypothalamic ceramide pathways may accelerate reproductive maturation, as proposed by Heras et al [64]. This mechanism, described in the context of excess adiposity, could also help contextualize the earlier pubertal signs observed in some girls in the PT group, despite their prepubertal gonadotropin levels.

### Strength, limitations, and future directions

The strength of this study lies in the rigorous selection of 184 girls with precocious puberty and PT using detailed clinical, biochemical, and radiological criteria, including DXA scans, a reliable tool for assessing bone mineral status and body composition in pediatric populations [65]. The comparison among the 3 groups (CPP/EP, PT, and PC) provided valuable insights into how obesity and body composition influence pubertal progression and bone mineral accrual. The use of BMAD and TBS, in addition to conventional BMD, adds clinical relevance by capturing complementary aspects of bone quality in pediatric populations. However, TBS could not be calculated in all patients, which represents a limitation of the study.

Limitations include the retrospective, observational, and cross-sectional design, precluding causality assessments, and the lack of evaluation of potential confounders such as socioeconomic, genetic, lifestyle, and environmental factors. Additionally, small subgroup sizes, particularly in girls with PC, limit generalizability. Another aspect to consider is the composition of the control group, which included prepubertal girls who had undergone DXA as part of previous research protocols or clinical evaluations for nonbone-related conditions; although strict exclusion criteria were applied, the nature of this recruitment may also have introduced bias. Another limitation is the heterogeneity of the PT group, which encompassed girls with premature breast development alone as well as those presenting with both thelarche and pubarche. This reflects the clinical overlap between PT and premature adrenarche and may have introduced variability in both hormonal and body composition parameters within the group. For this reason, we performed subgroup analyses comparing girls whose pubertal onset was marked by pubarche with those who presented with thelarche first, in order to better characterize this heterogeneity. In contrast, thelarche was the initial pubertal sign in almost all girls with CPP/EP, reflecting the intrinsic biological difference between the two cohorts: girls with CPP/EP showed clear evidence of central activation, whereas those with PT exhibited prepubertal gonadotropins and no signs of rapidly progressive puberty.

Given the retrospective nature of the study, some degree of misclassification cannot be completely excluded, particularly among PT cases who did not undergo GnRH stimulation testing; despite an initially reassuring evaluation, a small proportion could have represented very early or intermittent activation of the HPO axis. To mitigate this risk, girls were classified as PT without GnRH testing only when their clinical course and available investigations were consistently compatible with a nonprogressive phenotype.

Moreover, although the conventional cutoff of 8 years for breast development aligns with the Italian Society for Pediatric Endocrinology and Diabetology (ISPED) [66], international studies report earlier thresholds [67-71], particularly in non-European populations and in association with higher BMI and African American or Hispanic ethnicity. In our cohort, most girls were of European origin, with only 14.7% being non-European, which did not allow stratified analyses. As noted by Anderson et al [72], pubertal timing varies across ethnic groups, partly due to differences in body composition; therefore, our findings should be interpreted with caution and are most applicable to predominantly European populations.

Finally, while we hypothesize that girls in whom pubarche preceded thelarche may have had elevated adrenal androgens, consistent with what is described in premature adrenarche, the absence of specific androgen measurements prevents definitive classification and limits any causal interpretation regarding hormone–body composition relationships.

In conclusion, our findings suggest that early hormonal activation and adiposity may contribute to skeletal and body composition outcomes during puberty, highlighting the multifactorial nature of this process. Distinguishing between true CPP and PT remains clinically challenging, and DXA provides complementary information beyond hormonal and clinical evaluation. Both groups showed increased adiposity relative to controls, but girls with CPP exhibited a more advanced musculoskeletal profile, consistent with full activation of the hypothalamic–pituitary–gonadal axis. In contrast, the pattern observed in PT appeared more closely linked to adiposity and other peripheral influences on early pubertal signs, particularly in girls whose onset was marked by pubarche, who showed greater, and more centrally distributed fat, as reflected by their higher A/G ratio.

Since early pubertal timing has been associated with long-term adverse outcomes, tools that help characterize metabolic and skeletal profiles may be clinically relevant. Although DXA has no diagnostic role in CPP, it can provide complementary information on body composition and bone measures that may support clinical characterization and follow-up. The occurrence of pubarche preceding thelarche may seem reassuring, being observed in only 6.5% of CPP/EP cases vs 25% of girls with PT, yet these girls still showed relevant alterations in body composition. In this subgroup, DXA may support longitudinal assessment and individualized follow-up.

Regarding bone status, although BMD *Z*-scores were higher in CPP/EP and PT than in controls, this difference was not confirmed by BMAD, suggesting that the observed DXA signal is largely explained by differences in skeletal size rather than true volumetric densitometric gains.

More than 35% of total body and spinal BMC is accrued during the 3-4 years surrounding the onset of puberty in both males and females as [73]. Consequently, adolescence represents a critical period for skeletal development and fracture risk, underscoring the importance of investigating how early pubertal onset may affect bone health and contribute to long-term skeletal vulnerability.

Exploratory findings on TBS, which was lower in CPP/EP than PT, reinforce the need for longitudinal studies to clarify the clinical significance of both BMAD and TBS in this context.

## Data Availability

All datasets generated and analyzed during the current study are not publicly available but are available from the corresponding author on reasonable request.

## Abbreviations

A/G ratio: android to gynoid fat ratio
ALMI: appendicular lean mass index (appendicular lean mass/height²)
BA: bone age
BMAD: bone mineral apparent density
BMC: bone mineral content
BMD: bone mineral density
BMI: body mass index (kg/m²)
CA: chronological age
CPP: central precocious puberty
DXA: dual-energy X-ray absorptiometry
E2: estradiol
EP: early puberty
FFM: fat-free mass
FFMI: fat-free mass index (fat-free mass/height²)
FM: fat mass
FMI: fat mass index (fat mass/height²)
FSH: follicle-stimulating hormone
H: height
HPO axis: hypothalamic–pituitary–ovarian axis
IGF-1: insulin-like growth factor-1
L1-L4: lumbar spine
LH: luteinizing hormone
PC: prepubertal controls
PT: premature thelarche
SDS: standard deviation score
TBLH: total body less head
TBS: trabecular bone score
TH: target height
ΔBA – CA: bone age minus chronological age
ΔH – TH: height minus target height
